# A Comparison of Tissue Engineering Scaffolds Incorporated with Manuka Honey of Varying UMF

**DOI:** 10.1155/2017/4843065

**Published:** 2017-02-23

**Authors:** Katherine R. Hixon, Tracy Lu, Sarah H. McBride-Gagyi, Blythe E. Janowiak, Scott A. Sell

**Affiliations:** ^1^Department of Biomedical Engineering, Parks College of Engineering, Aviation, and Technology, Saint Louis University, St. Louis, MO, USA; ^2^Department of Orthopaedic Surgery, Saint Louis University, St. Louis, MO, USA; ^3^Department of Biology, Saint Louis University, St. Louis, MO, USA

## Abstract

*Purpose*. Manuka honey (MH) is an antibacterial agent specific to the islands of New Zealand containing both hydrogen peroxide and a Unique Manuka Factor (UMF). Although the antibacterial properties of MH have been studied, the effect of varying UMF of MH incorporated into tissue engineered scaffolds have not. Therefore, this study was designed to compare silk fibroin cryogels and electrospun scaffolds incorporated with a 5% MH concentration of various UMF.* Methods*. Characteristics such as porosity, bacterial clearance and adhesion, and cytotoxicity were compared.* Results*. Pore diameters for all cryogels were between 51 and 60 *µ*m, while electrospun scaffolds were 10 *µ*m. Cryogels of varying UMF displayed clearance of approximately 0.16 cm for* E. coli* and* S. aureus*. In comparison, the electrospun scaffolds clearance ranged between 0.5 and 1 cm. A glucose release of 0.5 mg/mL was observed for the first 24 hours by all scaffolds, regardless of UMF. With respect to cytotoxicity, neither scaffold caused the cell number to drop below 20,000.* Conclusions*. Overall, when comparing the effects of the various UMF within the two scaffolds, no significant differences were observed. This suggests that the fabricated scaffolds in this study displayed similar bacterial effects regardless of the UMF value.

## 1. Introduction

The medicinal use of honey dates back to 2100–2000 BC as documented on a Sumerian tablet. Its use primarily included treatment for the acceleration of wound healing. More recently, honey has reemerged due to the increasing prevalence of drug-resistant bacterial strains and has been utilized for the treatment of ulcers, burns, bed sores, and infections. Honey has been shown to possess antibacterial activity and provide a moist environment with high viscosity, forming a protective barrier [1]. It is primarily composed of sugars and water, as well as vitamins such as B complex and vitamin C. In total, more than 181 elements make up honey including amino acids, proteins, phenol antioxidants, flavonoids, nitric oxide (NO) metabolites, carotenoid-derived compounds, and antibiotic-rich inhibine [2]. Through degradation via bodily fluids, the glucose in honey is broken down into hydrogen peroxide and gluconic acid. The hydrogen peroxide exhibits antiseptic properties while the gluconic acid helps to lower the pH to around 3.2–4.5 at the wound site. Together, these characteristics assist in bacterial inhibition and reduction of the wound size [3–7]. Reduced pH has also been shown to encourage angiogenesis, a critical component of wound healing [2, 8, 9]. In addition, honey has demonstrated the ability to stimulate proinflammatory cytokines and move wound healing past an extended chronic inflammatory phase [10].

Manuka honey (MH) is collected by honey bees from the* Leptospermum scoparium* shrub, which is indigenous to New Zealand. This specific honey has been shown to exhibit additional antibacterial properties that are attributed to the presence of methylglyoxal (MGO) [9–11]. MGO is derived by nonenzymatic conversion of dihydroxyacetone which is present at high levels in the* L. scoparium* flower's nectar [12]. The levels of MGO highly contribute to the Unique Manuka Factor (UMF) of MH [13]. The UMF varies between batches of MH as affected by variables including the environment, processing, etc. [14]. After processing, the honey's UMF is tested and the antimicrobial efficacy is rated where 0 is low and 20 is high. The efficacy rating is meant to indicate the antibacterial potency. Such a measurement is based on the prohibition of bacterial growth by the honey in relation to a phenol positive control. For example, a 10 UMF would imply an antimicrobial potency of 10% phenol [15]. While other factors such as sugar and hydrogen peroxide have antibacterial activity, the UMF has been identified as the active ingredient in MH [9, 11, 13, 16, 17].

The field of tissue engineering utilizes scaffolds as a template to promote new tissue growth. The scaffold provides a network analogous to the natural extracellular matrix of the tissue. This tissue-specific architecture allows for ingrowth of cells and, eventually, complete replacement of the native tissue [18]. Two scaffold fabrication techniques were chosen for the incorporation of MH and were subsequently characterized. Electrospinning is one scaffold fabrication technique which leads to the production of polymeric, nonwoven meshes [19]. For this method, a polymer dissolved in a highly volatile solution is extruded through a syringe with a blunt-tip, conductive needle. As a high voltage is applied to the needle, the fluid is pushed through the tip in the form of a Taylor cone. The solvent evaporates off, and the polymer travels the distance from needle tip to collecting target (working distance), landing on a grounded collecting mandrel. The fibers form a porous, nonwoven network, as affected by parameters such as the polymer concentration, voltage, flow rate, and working distance [20–22].

While electrospinning has become commonplace in tissue engineering, there is only a small body of literature focused on the incorporation of honey into electrospun scaffolds. A previous study by Arslan et al. [23] fabricated electrospun composite scaffolds of both polyethylene terephthalate (PET)/honey and PET/chitosan/honey. Upon the incorporation of increasing concentrations of up to 40% honey, taken from flowers in the Meydancık countryside of the Artvin province, smooth and uniform fibers were obtained. MTT assay results demonstrated that the electrospun scaffolds incorporated with honey had no toxic effect on the cells; however, cells cultured with extracts of PET/honey (10% and 40%) were less confluent with altered morphology. A study by Maleki et al. electrospun poly(vinyl alcohol) (PVA) and Iran-Tabriz honey at different ratios [24]. This yielded uniform and smooth nanofibers with fiber diameter decreasing from 446 nm to 220 nm as honey incorporation increased to a 60/40 ratio. A study by Minden-Birkenmaier et al. [25] examined electrospun poly(*ε*-caprolactone) (PCL) incorporated with 1, 5, 10, and 20% v/v MH. The elasticity and strength of the scaffolds decreased with the addition of honey but did not affect the degradation rate. Additionally, the incorporation of MH increased fibroblast proliferation and exhibited clearance of both a model Gram-positive bacterium,* Streptococcus agalactiae* (Group B* Streptococcus*), and a model Gram-negative bacterium,* Escherichia coli*.

The second scaffold fabrication technique chosen for evaluation, cryogels, involves the controlled freezing of a polymer solution which leads to the formation of ice crystals throughout the structure [26]. When thawed, the ice melts out of the scaffold, leaving a macroporous, spongy, and mechanically durable structure [27–29]. Similar to electrospinning, cryogel scaffolds are gaining popularity in the realm of tissue engineering, but there is little literature on their integration with honey. In a previous study by Kadakia et al. [30], the addition of MH to silk fibroin (SF) cryogels resulted in decreased pore size from 151 *µ*m in plain SF cryogels to 124 and 78 *µ*m for 1 and 5% MH, respectively. However, all MH-containing cryogels exhibited cellular attachment of MG-63 cells by day 7 with some infiltration after 28 days. In previous unpublished research from our laboratory, we have also been able to incorporate 1, 5, and 10% MH in both SF and gelatin cryogels. These cryogel scaffolds were then evaluated for the impact of MH inclusion on their physical properties, as well as bacterial clearance and glucose release. Similarly, the swelling potential and porosity decreased with increasing concentrations of incorporated MH, but full infiltration of MG-63 cells occurred after 28 days. All cryogels incorporated with MH resulted in clearance of* Escherichia coli*,* Streptococcus agalactiae*, and* Staphylococcus aureus* over a 24-hour period. Additionally, a burst release of glucose was measured after one hour, with a smaller, continued release continuing over two weeks (unpublished data). While this study similarly examined MH in cryogel scaffolds, only a single UMF was incorporated and characterized.

While honey has previously been incorporated into various tissue engineered scaffolds, no study to date has compared the effects of different UMF on MH's properties when slowly released from a tissue engineering scaffold. This study evaluated the incorporation of low and high UMF MH into both SF electrospun and cryogel scaffolds. SF is a natural, biocompatible polymer chosen for its superior mechanical properties to all other natural polymers and a majority of synthetic materials. Additionally, SF contains cell attachment sites and has previously been used for both the fabrication of electrospun and cryogel scaffolds [31–37]. Both types of SF scaffolds were tested in parallel to determine the effect of various UMF on porosity and cytotoxicity, as well as bacterial clearance and adhesion. It is anticipated that, as was previously shown with bolus MH, an increase in the UMF will yield an increase in bacterial clearance.

## 2. Materials and Methods

### 2.1. Formation of Scaffolds

#### 2.1.1. SF Cryogel (CG) Scaffold

The aqueous SF for CG fabrication was prepared as specified previously [38]. Briefly, 5% 5 or 20 UMF MH (Manuka Honey, UMF 5+, 10+, 15+, and 20+, Melita, New Zealand; Manuka Honey, Medical Grade 12+, Ndal Laboratories, California) was dissolved in the SF solution using a mechanical spinner. Once the solution was cooled to 4°C, 0.5 mL of this solution (4.5% w/v) was placed in 2 mL rounded bottom microcentrifuge tube. For fabrication of the CG, the tube was placed in a slightly larger beaker filled with ice water. A sonication probe (probe intensity of 2, Fisher Sonic Dismembrator Model 100) was lowered into the solution and sonicated for 30 seconds. Once finished, the tube was removed, tapped to remove bubbles, and transferred to a −20°C stirred methanol bath. After 24 hours of freezing, the tubes were thawed in room temperature (RT) deionized (DI) water for an additional 24 hours [30]. Three of each CG scaffold were used for all characterization and testing.

#### 2.1.2. SF Electrospun (ES) Scaffold

To create the solution, 5% of 5 and 20 UMF MH were, separately, dissolved in 1,1,1,3,3,3-hexafluoro-2-propanol (HFP, Oakwood Chemical) through a 20-minute sonication in a RT water bath (Branson 200 Ultrasonic Cleaner, Branson Ultrasonics). Lyophilized SF (5%) was then dissolved overnight in the MH-containing solution using a mechanical shaker. The solution was loaded into a 5 mL syringe tipped with a blunted 18-gauge needle (PrecisionGlide, Becton Dickinson). Electrospinning was completed using a syringe pump (78-01001, Fisher Scientific) set at 3 mL/hr. A high-voltage DC power supply (CZE1000PN30, Spellman High Voltage Electronics Corp.) provided a voltage of 23 kV onto a spinning (400 rpm) rectangular (0.5 cm × 2.5 cm × 9 cm) stainless steel mandrel using a working distance of 16.5 cm. All ES scaffolds were removed from the mandrel and stored at −20°C in a desiccation chamber. Three of each ES scaffold were used for all characterization and testing.

### 2.2. Scaffold Characterization

#### 2.2.1. Pore Analysis

*(1) Scanning Electron Microscope (SEM)*. All scaffolds were frozen at −80°C for one hour prior to lyophilization for 24 hours. Following this, the scaffolds were mounted on an aluminum stub and sputter coated (SoftComp, Bal-Tec SCD 005) in gold at 20 mA for 360 seconds. SEM (Zeiss, Evo LS15) images were taken at 500x and 2,000x for the CG and ES scaffolds, respectively, at an operating voltage of 5 kV. SEM images of the ES scaffolds were analyzed using Image J (NIH), providing both fiber and pore diameter (*µ*m). Briefly, the scale bar was set according to pixel size and the pore's long diameter and fiber diameter were measured.

*(2) Microcomputed Tomography (μCT)*. Both scaffold types were also analyzed using *µ*CT (*μ*CT 35, Scanco Medical, Wayne, PA) while hydrated. The central area of each scaffold was scanned. The CG scaffolds were scanned using the parameters of X-ray tube potential 45 kVp, X-ray intensity 4 W, isotropic voxel size 7 *μ*m, integration time 600 ms, frame averaging 1, projections 500, and medium resolution scan. The ES scaffolds were scanned using the parameters of X-ray tube potential 45 kVp, X-ray intensity 4 W, isotropic voxel size 7 *μ*m, integration time 600 ms, frame averaging 1, projections 500, and high resolution scan. Scaffold and pore geometry as well as volume were obtained using the manufacturer installed trabecular morphology analysis. Voxels above a threshold of 80 per milles (determined through pilot testing) were considered scaffold and those below 80 per milles were considered empty space.

#### 2.2.2. Bacterial Clearance

Overnight cultures containing brain heart infusion (BHI) were prepared with fresh isolates of either* Escherichia coli* K99 (*E. coli*; ATCC: PTA-5951) or* Staphylococcus aureus* subsp.* aureus* Rosenbach (*S. aureus*; ATCC: 12600).* E. coli* was chosen as a model Gram-negative bacterial strain, while* S. aureus* was chosen as a model Gram-positive bacterial strain; both bacterial strains are common wound pathogens. The overnight cultures of the bacteria were spread onto quad BHI plates using a sterile swab and sterile discs were placed in individual sections along with 5, 10, 12, 15, and 20 UMF MH. Additionally, 6 mm and 10 mm punches of the CG and ES scaffolds, respectively, were also placed in other sections of the dishes. Sterile discs and MH were used as controls. All plates were incubated at 37°C for 24 hours at which time images were taken of each plate. The full and partial clearance of the images was analyzed using Image J (NIH) by setting the scale bar with respect to the image pixel size. The distance across the clearance was measured along with the diameter of the scaffold or sterile disc. The scaffold/disc diameter was subtracted from the total clearance and divided by two to calculate the distance cleared away from the scaffold or disc (cm).

#### 2.2.3. Bacterial Adhesion

The scaffolds were incubated in bacteria to analyze the effects of MH on bacterial adhesion, as optimized from a previous paper [39]. An overnight culture of* S. aureus* was created using 50 mL of BHI. This bacterial solution was serially diluted and plated to quantify the starting colony forming units/ml (CFU/ml). Each type of scaffold was placed in 2 mL of the bacterial solution in an untreated 24-well plate (Fisher Scientific, New Hampshire). The plates were then placed at 37°C for 4 hours with constant swirling. After the incubation period, all scaffolds were rinsed twice with PBS to remove any nonadherent microorganisms. Some of each type of scaffold were removed and placed in formalin at 4°C. These scaffolds were dehydrated in graded alcohol (30, 50, 70, 80, and 90% for 15 minutes and 100% for 1 hour) and underwent critical point drying (CPD 030 Critical Point Dryer). The scaffolds were then sputter coated with gold and SEM images were taken at 10,000x at an operating voltage of 15 kV. All other scaffolds were moved to a microcentrifuge tube and submerged in 1 mL of fresh, sterile PBS. The tubes were then continuously vortexed for 30 minutes at RT to elute the microorganisms from the surface of the scaffold. Aliquots of 100 *µ*L of the solution were serially diluted on BHI agar plates to quantify the CFU/ml of the bacterial adhesion solution.

#### 2.2.4. Glucose Assay

All types of scaffolds were placed in 400 *µ*L of sterile PBS. The PBS was retained and replaced at 1 hour and days 1, 4, 7, and 14. The releasate of the soaked cryogels was assayed for glucose (Glucose Assay Kit, Sigma Aldrich) as an indicator of MH release.

#### 2.2.5. Cytotoxicity

The CG and ES scaffolds were placed in 400 *µ*L of DMEM/F-12 media supplemented with 10% fetal bovine serum (FBS) (Biowest, Texas) and 1% penicillin-streptomycin solution (Hyclone, Pennsylvania). The media was retained at 1 and 18 hours and days 1, 4, 7, and 14. 50,000 human dermal fibroblasts (hDF, passage 4; ATCC, Virginia) were seeded onto a 48-well plate and placed in the incubator at 37°C and 5% CO_2_ for 2 hours to allow for full attachment. The cells were then fed with 200 *µ*L of the conditioned DMEM/F12 combined with 200 *µ*L of fresh DMEM/F12 media. After 72 hours, an MTS assay (Celltiter 96 Aqueous Nonradioactive Cell Proliferation Assay, Promega) was used to quantify the number of cells in each well for all time points. To do this, the modified media were removed and replaced with 200 *μ*L of supplemented DMEM/F-12 media. Next, 40 *μ*L of MTS/PMS solution was added to each well and the plates were incubated for one hour at 37°C. A 100 *µ*L aliquot was removed and analyzed at 490 nm (SpectraMax i3 plate reader, Molecular Devices) to quantify cell number as affected by each condition.

#### 2.2.6. Statistical Analysis

For all tests, statistical analyses were performed using IBM SPSS software with a statistical significance determined at an alpha value of 0.05. All groups were analyzed using a one-way ANOVA with a Tukey post hoc analysis to evaluate the significance.

## 3. Results and Discussion

### 3.1. Pore Analysis

#### 3.1.1. Scanning Electron Microscope (SEM)

**Figure 1:**
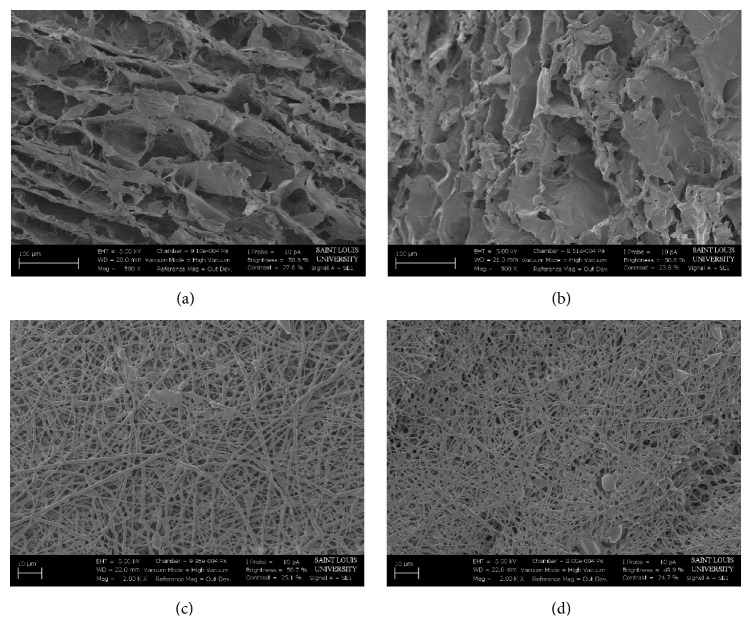
SEM images taken at 500x of (a) 5 and (b) 20 UMF CG scaffolds. SEM images taken at 2,000x of (c) 5 and (d) 20 UMF ES scaffolds.

SEM imaging was employed to analyze the surface topography and pore structure (Figure 1). Visually, the CG pores were open and varied in size. This nonuniformity is most likely due to the sonication method of fabrication. This can result in the formation of bubbles as well as the pores formed by the ice crystals [40]. The ES scaffolds exhibited small clumps or beads which are most likely due to MH that was not included within the polymer fiber matrix [23]. Visually, the ES scaffolds had a very similar fiber distribution and size between both 5 and 20 UMF MH. However, Image J analysis found that the average pore diameter of 5 UMF was significantly larger than 20 UMF (Table 1). There was no significant difference between fiber diameters of the ES scaffolds (*p* < 0.05).

#### 3.1.2. Microcomputed Tomography (*μ*CT)

**Figure 2:**
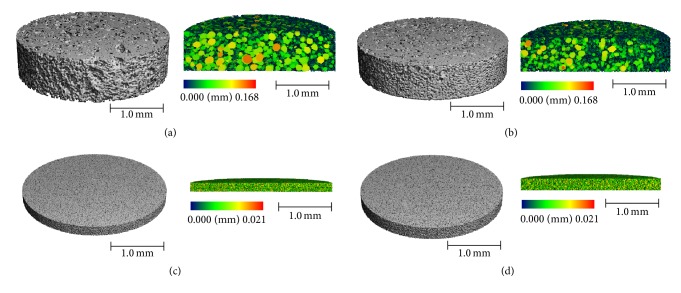
*µ*CT 3D reconstruction of 5 and 20 UMF (a, b) CG scaffolds, as well as 5 and 20 UMF (c, d) ES scaffolds. A sagittal cross section of each is provided displaying the inner pores, where the color bar denotes the size of the pores.

**Figure 3:**
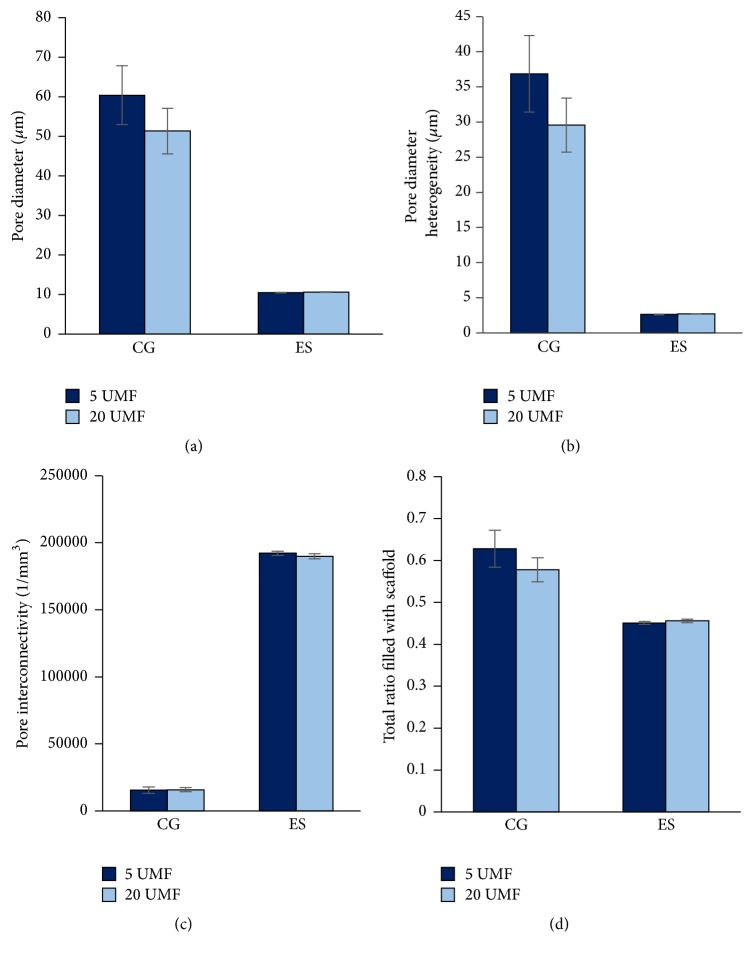
Measurements of (a) pore diameter and (b) pore heterogeneity as found through *µ*CT at a threshold of 80 per milles. The (c) pore connection density and the (d) total ratio of the overall scaffold that is filled with polymer were also demonstrated. There was no significant difference between 5 and 20 UMF incorporated into either CG or ES scaffolds (*p* < 0.05).

*µ*CT was used to provide a quantitative analysis of the cryogel scaffold porosity (Figure 2). While Image J (NIH) analysis is commonly used, this technique only provides information regarding the surface of the cryogel scaffold and not the interior. A previous study supported that such measurements are likely not as accurate compared to a 3D measurement such as *µ*CT [29]. The 5 and 20 UMF CG scaffolds had an average pore diameter of 60 and 51 *µ*m, respectively. The 5 and 20 UMF ES scaffolds both had an average pore diameter of 10 *µ*m (Figure 3(a)), similar to the measurements found using Image J (Table 1). Previous literature has identified a 100 *µ*m pore size as necessary for cell infiltration and angiogenesis to occur [41]. A high porosity is also necessary for the diffusion of nutrients and growth factors, and while these pores were not as large, the size was fairly consistent throughout the structure [42]. As observed from the SEM images, both types of CG scaffolds had a high level of heterogeneity of the pores, while relative smaller pore sizes were observed for the ES scaffolds (Figure 3(b)). Both the 5 and 20 UMF CG had a connection density of approximately 16,000 1/mm^3^. Additionally, both 5 and 20 UMF ES scaffolds had a high connection density of approximately 190,000 1/mm^3^ (Figure 3(c)). Lastly, both the CG and ES scaffolds had similar ratios of scaffold to the entire structure (Figure 3(d)). Statistically, there was no difference within a scaffold type upon the incorporation of 5 or 20 UMF MH (*p* < 0.05). Thus, the incorporation of these various MH UMF did not affect the scaffolds' porosity and interconnectivity.

### 3.2. Bacterial Clearance

**Figure 4:**
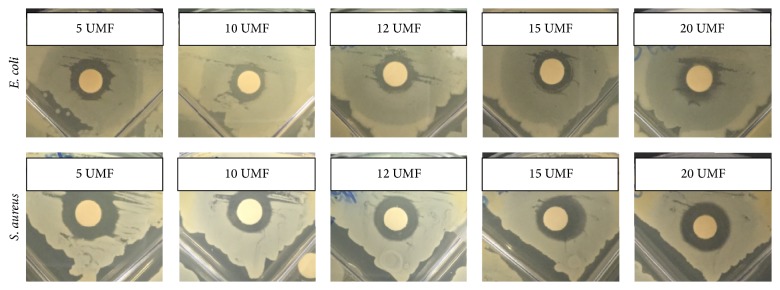
Representative images of the clearance of* E. coli* and* S. aureus* by 5, 10, 12, 15, and 20 UMF MH.

**Figure 5:**
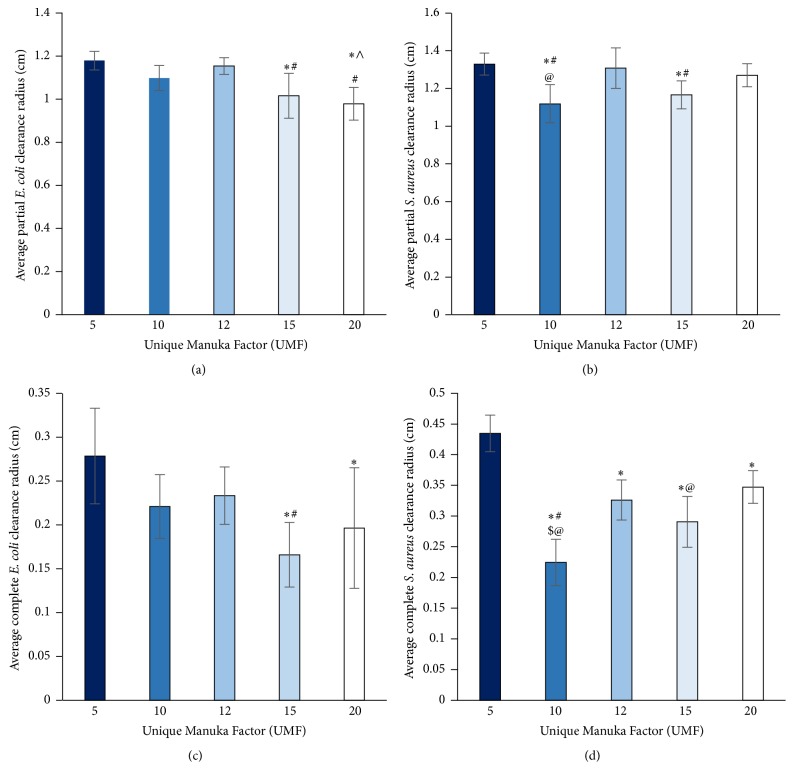
Partial and complete clearance radius of (a, c)* E. coli* and (b, d)* S. aureus* by various UMF honey bolus. *∗* and ∧ denote clearance that is significantly smaller than 5 and 10 UMF, respectively. # denotes a clearance that is significantly smaller than 12 UMF. $ and @ denote clearance that is significantly smaller than 15 and 20 UMF, respectively (*p* < 0.05).

**Figure 6:**
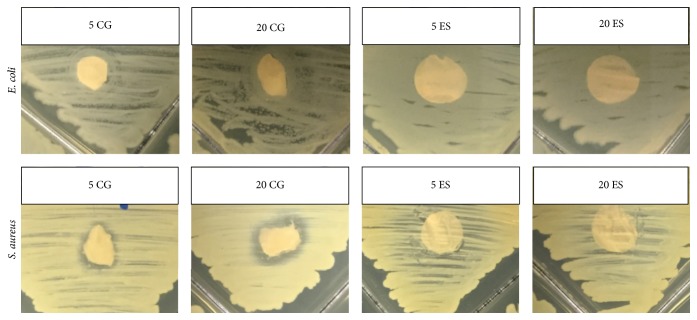
Representative images of the clearance of* E. coli* and* S. aureus* by 5 and 20 UMF CG and ES scaffolds. Both 5 and 20 UMF bolus honey as well as sterile discs were used as controls (not pictured).

**Figure 7:**
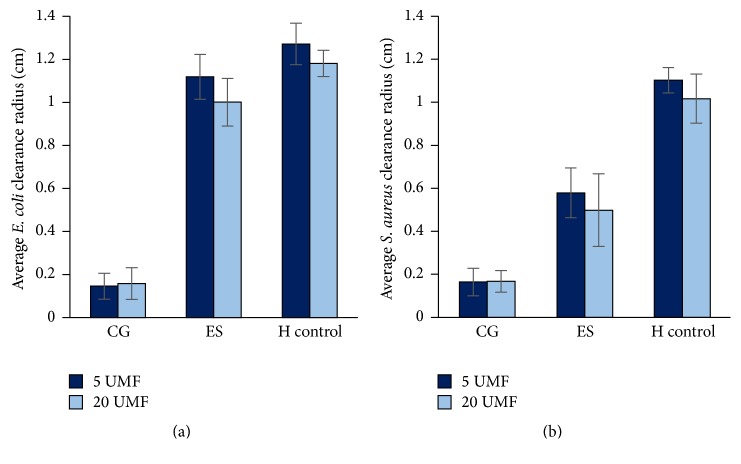
Partial clearance radius of (a)* E. coli* and (b)* S. aureus* by various MH UMF incorporated into CG and ES scaffolds, as well as UMF honey bolus control. Note that there was no significant difference in clearance by the 5 and 20 UMF when incorporated into CG or ES scaffolds (*p* < 0.05).

Five different UMF of MH were analyzed for bacterial clearance on both* E. coli* and* S. aureus* over 24 hours (Figure 4). Both the complete clearance and the partial clearance were measured as effected by the bolus of honey (Figure 5). Note that complete clearance occurred when no bacteria remained while partial clearance exhibited less bacteria in comparison to the lawn of bacteria. All types of UMF exhibited similar amounts of clearance around the discs with boluses of honey. However, for the partial clearance of* E. coli*, 5 UMF had significantly larger clearance than 15 and 20 UMF, 10 UMF had significantly larger clearance than 20 UMF, and 12 UMF had significantly larger clearance than 15 and 20 UMF. For complete clearance of* E. coli*, both 5 and 12 UMF had significantly larger clearance than 15 UMF. The 5 UMF was also significantly larger than 20 UMF. With respect to the partial clearance of* S. aureus*, both 5 and 12 UMF had significantly larger clearance than 10 and 15 UMF. A UMF of 20 was also significantly larger than 10 UMF. Lastly, for the complete clearance of* S. aureus*, 5 UMF had significantly larger clearance than all other UMF, and 10 UMF had significantly smaller clearance than all other UMF. A UMF of 20 was also significantly larger than a UMF of 15 (*p* < 0.05). As the UMF number has previously been shown to be indicative of the antimicrobial efficacy, it is interesting that, in many instances, 5 UMF resulted in larger clearance than the higher valued UMF. Thus, for the incorporation into scaffolds, both a high value and low value UMF (5 and 20) were chosen for comparison. When incorporated into both CG and ES scaffolds, the clearance was much smaller due to the sustained release of MH from the scaffold (Figures 6 and 7). The 5 and 20 UMF CG had clearance of* E. coli* of approximately 0.15 and 0.16 cm, respectively. In comparison, they had clearance of* S. aureus* of approximately 0.16 and 0.17 cm, respectively. The 5 and 20 UMF ES scaffolds had larger* E. coli* clearance of 1.12 and 1.00 cm, whereas they had* S. aureus* clearance of 0.58 and 0.50 cm, respectively. The 5 and 20 UMF bolus control had clearance of over 1 cm for both types of bacteria. For both types of bacteria clearance by scaffolds, there was no significant different between the 5 and 20 UMF CG scaffolds or the 5 and 20 UMF ES scaffolds (*p* < 0.05). This demonstrates that when either 5 or 20 UMF MH is incorporated into various types of scaffolds, their clearance of both* E. coli* and* S. aureus* does not differ.

### 3.3. Bacterial Adhesion

**Figure 8:**
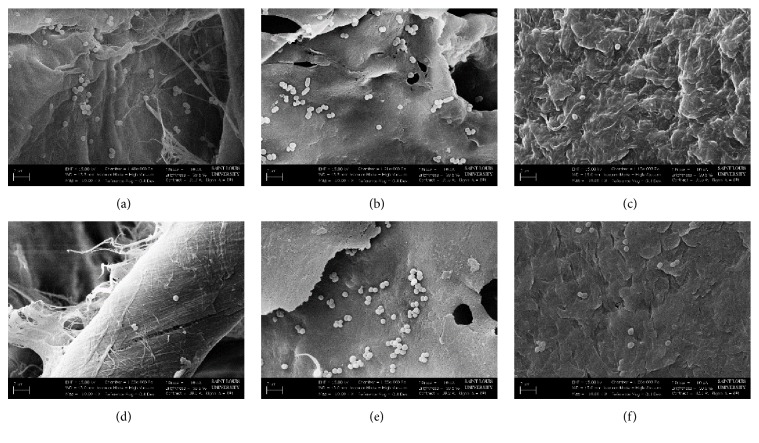
SEM images taken at 10,000x of* S. aureus* adhesion to the (a) sterile disc and (d) 5 mg vancomycin disc controls, (b) 5 and (e) 20 UMF CG, and (c) 5 and (f) 20 UMF ES.

**Figure 9:**
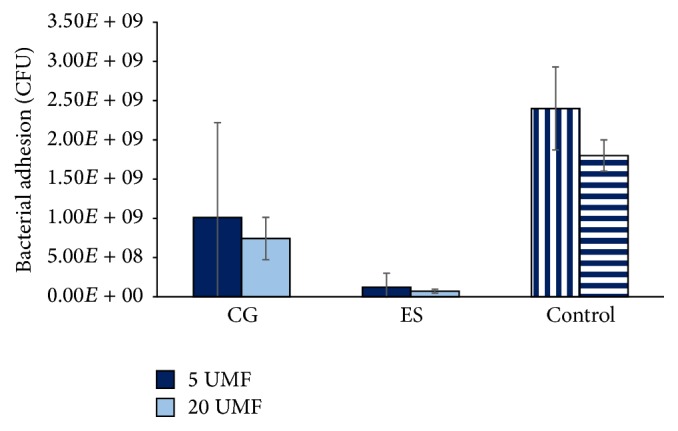
Bacterial adhesion of* S. aureus* to both CG and ES scaffolds as effected by the incorporation of 5 and 20 UMF MH. Note that the sterile disc control (left) is denoted by vertical bars and the vancomycin disc control (right) is denoted by horizontal bars. There was no statistical difference within each scaffold type upon the addition of either 5 or 20 UMF. Both the sterile disc and vancomycin disc control had significantly more adhesion than 5 and 20 UMF ES. Also, the sterile disc was significantly larger than 20 CG (*p* < 0.05).

All scaffolds and controls were examined for bacterial adherence where both a sterile disc and a disc containing 5 mg vancomycin (BD, New Jersey) served as the controls (Figure 8). Here,* S. aureus* can be noted on both control discs (Figures 8(a) and 8(d)). Additionally, the bacteria were found throughout the pores of the CG (Figures 8(b) and 8(e)).* S. aureus* can also be seen on the surface of the ES scaffolds, but visually to a lesser degree (Figures 8(c) and 8(f)). The critical point drying caused the ES fibers to condense and, thus, the SEM was only able to capture the bacteria that remained on the very surface. To provide a quantitative measurement of bacteria adhered to and throughout the scaffolds and discs, the adhesion of* S. aureus* was measured through CFU/ml (Figure 9). Note that all data was normalized by mass, due to the various porosities of the scaffolds. Both scaffold types incorporated with MH had less bacterial adherence than the sterile and vancomycin disc controls. Specifically, there was no statistical difference between 5 and 20 UMF CG or between 5 and 20 UMF ES. However, the disc control was significantly larger than both ES scaffolds and 20 CG. The vancomycin disc control was also significantly larger than both ES scaffolds (*p* < 0.05).

### 3.4. Glucose Assay

**Figure 10:**
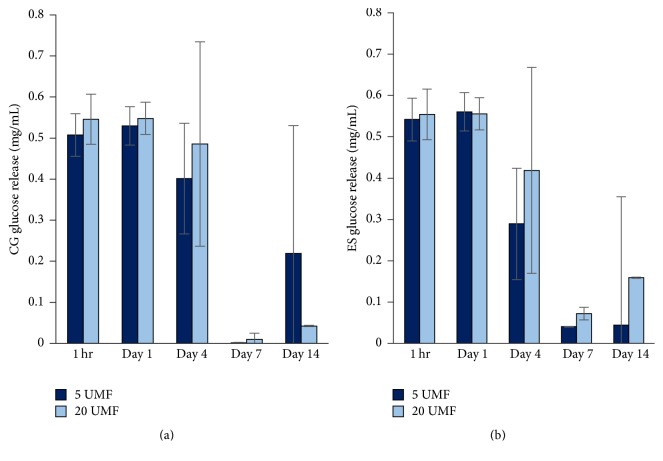
Glucose release from CG and ES scaffolds incorporated with 5 and 20 UMF MH at various time points. No statistical significance was found (*p* < 0.05).

The release of glucose was measured over 14 days (Figure 10). Both the CG and ES scaffolds released approximately 0.5 mg/mL of glucose for the first 24 hours. After 4 days, the release slightly decreased and by days 7 and 14 the release was much smaller in both 5 and 20 UMF. This demonstrates the sustained release of glucose as the scaffolds break down. There was no significant difference between any of the scaffolds, regardless of the type or which UMF was incorporated (*p* < 0.05). This shows that the glucose is being released by the scaffolds in similar amounts and over an extended period of time for both 5 and 20 UMF CG and ES scaffolds.

### 3.5. Cytotoxicity

**Figure 11:**
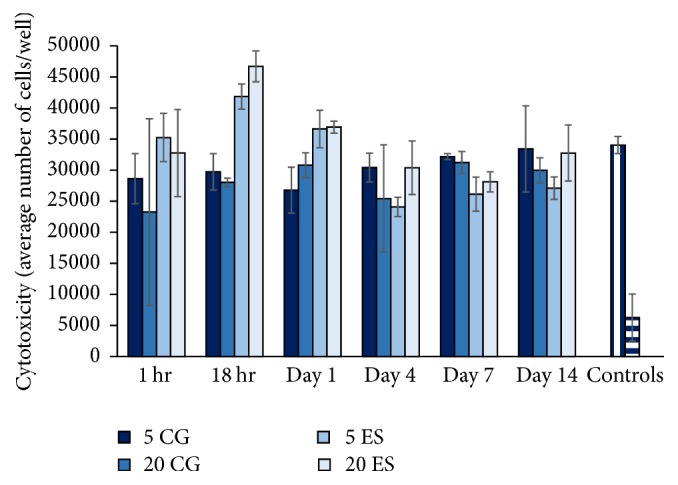
Cell proliferation as affected by 5 and 20 UMF CG and ES scaffold conditioned media. Note that, for the controls, regular media (left) were denoted by vertical bars and 5% MH media (right) were denoted by horizontal bars. There was no significant difference between 5 and 20 UMF within each specific type of scaffold (*p* < 0.05).

The retained media were placed on fibroblasts for 3 days (Figure 11). Overall, the MH media from both CG and ES scaffolds did not largely effect cell proliferation; none of the cell numbers dropped below 20,000. In comparison, when 5% MH media were placed on the cells, the number of cells dropped to around 5,000. This demonstrates the cytotoxic effects of MH when placed directly on the cells without incorporation into a scaffold. At 18 hours, both ES scaffolds had significantly more cells than both CG scaffolds. At day 1, 5 CG had significantly less cells than both ES scaffolds. At day 7, 5 ES had significantly less cells than both CG scaffolds. Despite this, there was no significant difference between cell numbers within a scaffold type upon the incorporation of 5 or 20 UMF MH (*p* < 0.05). Previous studies have shown that concentrations of MH, as low as 5%, are cytotoxic in vitro. This is hypothesized to be due to an acidic pH in a closed-off environment [13]. This is further supported by the MH control seen in Figure 11. However, this study suggests that when MH is incorporated into scaffolds, there is no cytotoxic effect. Such an outcome is most likely due to the sustained release of glucose seen in Figure 10, where the release of MH is slow enough that it is not extremely cytotoxic to the cells.

## 4. Conclusions

Medicinal honey has been used for wound healing for centuries. MH is a unique type of honey, from New Zealand, which possesses potent antibacterial properties due to a special inherent UMF. This UMF is assigned to the honey based on its antimicrobial efficacy. Despite this, no current study has compared the various UMF of MH as it effects bacterial clearance when incorporated into a tissue engineering scaffold. Within a scaffold type (CG or ES), there was no significant difference in porosity, bacterial clearance and adhesion, glucose release, or proliferation of cells as effected by the incorporation of 5 versus 20 UMF MH. This suggests very little difference between the antimicrobial efficacy of the various UMF. Instead, the tissue engineered scaffolds demonstrated the same properties and effects on bacteria, regardless of a high or low UMF value. Note that this result is based solely on in vitro data and the overall effect may be different in an in vivo environment. However, such an outcome is most likely due to the sustained release of the MH from the scaffolds as shown in the glucose assay. Consequently, when MH is released at this slow rate, the difference in UMF has no impact on bacterial clearance and adhesion. Thus, a tissue engineered scaffold could be incorporated with MH of any UMF, resulting in the same bactericidal outcome. Such a scaffold incorporated with MH of any UMF would have a use in a number of applications, especially wound healing.

## Figures and Tables

**Table 1:** Pore and fiber diameter of the ES scaffolds by Image J.

	Pore diameter (*µ*m)	Fiber diameter (*µ*m)
5 UMF	9.17 ± 3.60	0.97 ± 0.31
20 UMF	6.17 ± 2.42	1.01 ± 0.30
